# Strategies for improving the production of bio-based vanillin

**DOI:** 10.1186/s12934-023-02144-9

**Published:** 2023-08-05

**Authors:** Ying Liu, Lichao Sun, Yi-Xin Huo, Shuyuan Guo

**Affiliations:** 1 Key Laboratory of Molecular Medicine and Biotherapy, School of Life Science, Beijing Institute of Technology, Beijing, 100081 China; 2 Beijing Institute of Technology (Tangshan) Translational Research Center, Hebei, 063611 China

**Keywords:** Vanillin, Biosynthesis, Chassis development, Pathway construction, Process optimization, Dynamic regulation

## Abstract

Vanillin (4-hydroxy-3-methoxybenzaldehyde) is one of the most popular flavors with wide applications in food, fragrance, and pharmaceutical industries. However, the high cost and limited yield of plant extraction failed to meet the vast market demand of natural vanillin. Vanillin biotechnology has emerged as a sustainable and cost-effective alternative to supply vanillin. In this review, we explored recent advances in vanillin biosynthesis and highlighted the potential of vanillin biotechnology. In particular, we addressed key challenges in using microorganisms and provided promising approaches for improving vanillin production with a special focus on chassis development, pathway construction and process optimization. Future directions of vanillin biosynthesis using inexpensive precursors are also thoroughly discussed.

## Background

Vanillin, also known as 4-hydroxy-3-methoxybenzaldehyde, is the major component of vanilla products. It is a popular flavoring compound with wide applications in food, fragrance, pharmaceutical, and chemical industries [1]. In particular, vanillin serves as a high-grade flavor additive in food industry with high sweetness intensity and creamy vanilla taste [2]. Moreover, it is applied as a biological preservative owing to the antibacterial, anti-mutagenic and antioxidant activities [3]. Synthetic vanillin is also utilized in the production of household products such as deodorants, air fresheners, floor polish, and herbicides. Currently, vanillin has a market demand of approximately 20,000 tons, while only less than 2,000 tons are naturally available [4].

Traditional production methods of vanillin include plant extraction and chemical synthesis. Typically, plant extraction method uses ethanol as a solvent to extract vanillin from vanilla pods. The time-consuming process of conventional cultivation, harvesting, and maintenance of vanilla plants, along with the extremely low yield, lead to a high price of natural vanillin and limit the industrial application [5]. Plant cell culture technique that was introduced by Knuth et al. in 1991, was performed by inducing callus formation from vanilla fragrans cut strips in a sterile dish, yielding 0.099 g/L vanillin [6]. The callus culture could be transferred to a fresh medium for further cultivation, offering a promising alternative to traditional extraction methods. However, this technology has not yet yielded satisfactory results due to the low yield [7]. Currently, 95% of vanillin in the market is produced by chemical synthesis using lignin and guaiacol as the main materials. By hydrolyzing lignin under alkaline conditions followed by oxidation, vanillin could be obtained with a yield of approximately 10% [8]. The Riedel reaction has been used to combine guaiacol with glyoxylic acid to synthesize 3-methoxy-4-hydroxymandelate sodium, which could be further oxidized and acidified to produce vanillin [9]. However, the chemical synthesis has several disadvantages, including a complex process, expensive raw materials and high energy consumption along with poor substrate specificity, low stereoselectivity and high pollution. Moreover, the amount of synthetic vanillin used in food is strictly limited by the European Expert Committee.

Growing demands for sustainability and natural vanillin have renewed interest in developing alternative, low-cost and bio-based methods. Bioconversion methods mimic the way that plants produce vanillin by utilizing enzymatic/microbial catalysts and inexpensive natural substrates, thus meets the food safety requirements of the European Union (EU), China Food and Drug Administration (CFDA), and U.S. Food and Drug Administration (FDA) [10]. These production methods yield bio-based vanillin at mild conditions with high efficiency and specificity, low energy consumption, and little pollution. For example, vanillyl alcohol oxidase (VAO) has been used as an enzyme catalyst for oxidizing creosote from wood tar alcohol to vanillin, reaching a conversion rate of nearly 100% [11]. Specifically, microbial synthesis of vanillin from inexpensive substrates is of highly interest due to the sustainable and cost-effective manner [12]. To highlight the current state and potential of microbial production, a comprehensive discussion will be provided by addressing the main bottlenecks and various feasible strategies will be put forward from the perspectives of chassis development, pathway construction and process optimization.

## Developing robust production chassis

**Fig. 1:**
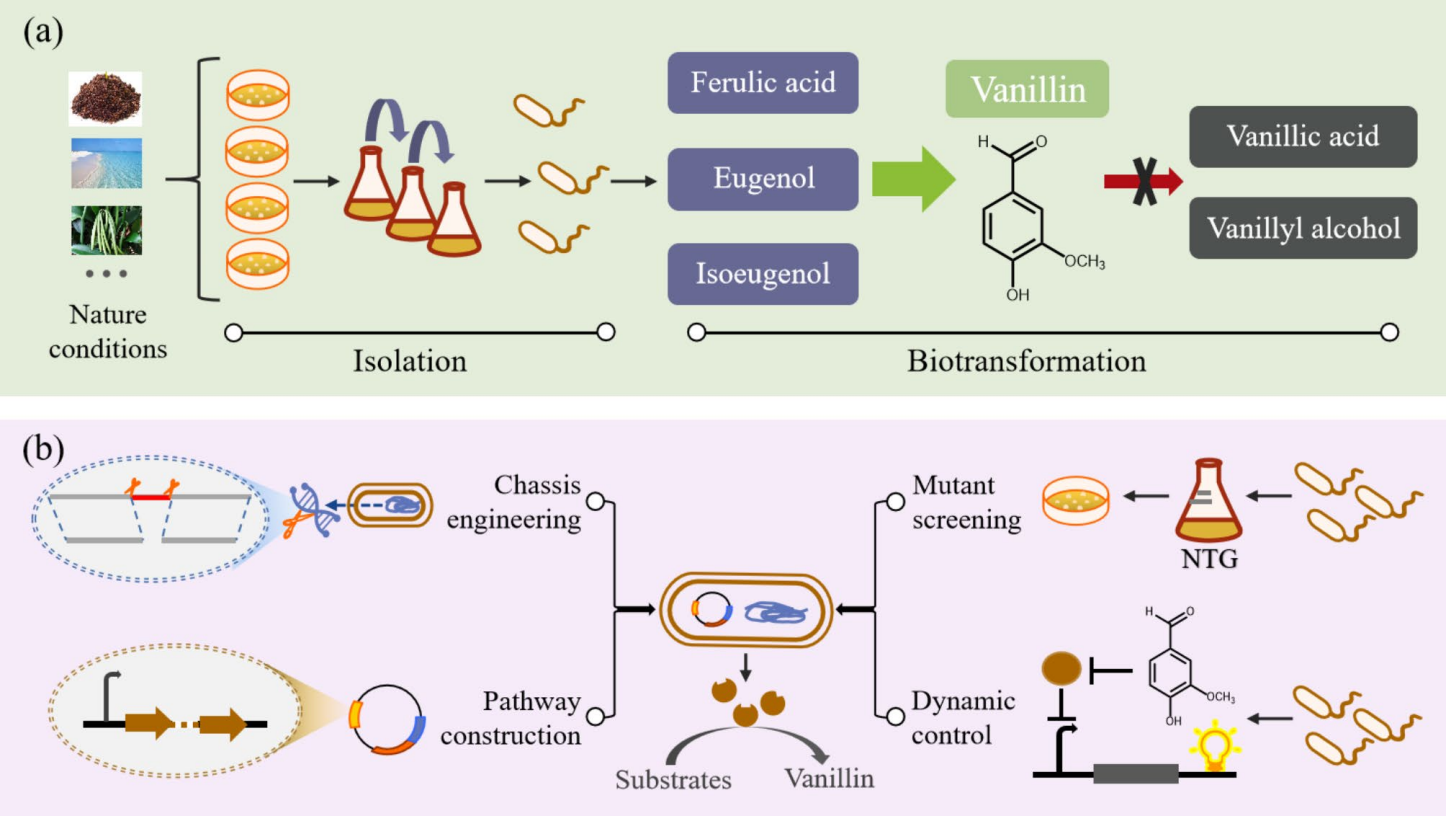
Development and modification of vanillin production chassis. (**a**) Selection and improvement of the natural chassis. (**b**) Construction and optimization of the recombinant chassis

Some natural microorganisms have the ability to synthesize low amount of vanillin, however, the processes are confronted with common challenges such as low conversion efficiency, high degradation rate and product cytotoxicity, limiting the application in large-scale production. To address these, approaches such as strain screening and laboratory evolution have been employed to achieve optimized metabolism (Fig. 1a). Despite this, undomesticated properties of natural hosts, such as poor transformation efficiency of exogenous DNA [13], rendered them difficult to be engineered. An alternative is to explore the potential of conventional model hosts such as *Escherichia coli* and *Saccharomyces cerevisiae* in the biotechnological production. In this regard, methods such as high-throughput screening and genomic engineering could be applied to improve the chassis performance (Fig. 1b).

### Screening and improving the natural chassis

Microorganisms such as *Bacillus* [14], *Pseudomonas* [15], *Streptomyces* [16], and some wild fungi [17] naturally utilize substrates such as isoeugenol, eugenol and ferulic acid to produce vanillin. Early studies mainly focused on the identification and isolation of strains from natural environments such as seawater, soil, and industrial effluents. Specifically, one-step plate screening [14], two-step screening using culture medium and agar plate [18], and enrichment technique [19]have been used to screen strains capable of utilizing a specific substrate as the sole carbon source and energy source. During this process, strain with better substrate tolerance could also be screened out. For example, *Pseudomonas sp*. ISPC2 was isolated from soil and was able to convert 10 g/L isoeugenol to form 1.15 g/L vanillin in 96 h [19]. Similarly, a soil-derived *Bacillus pumilus* S-1 was screened out, the resting cells generated 3.75 g/L vanillin from isoeugenol over 150 h with a molar yield of 40.5% [18]. Beyond bacteria, yeasts such as *Candida galli* PG06 [20] and *Trichosporon asahii* MP24 [21] have also been isolated, converting isoeugenol to vanillin with a titer in the range of 1.12 g/L to 2.40 g/L. By using enriched culture technique, a newly isolated *Pseudomonas resinovorans* SPR1 utilizing eugenol was obtained, producing 0.24 g/L vanillin over 30 h [22]. *Debaryomyces hansenii* MTCC 539 was identified to be able to rapidly convert ferulic acid to 4-vinylguaiacol, which was further oxidized to vanillin and vanillic acid, however, the maximum concentration of vanillin does not exceed 169 mg/L [23].

Although the natural chassis harboring the native biosynthetic pathway is superior in substrate and vanillin tolerance, the conversion ability is usually reduced with the increase of substrate concentration. For example, *Bacillus subtilis* B7 was able to grow at a range of 0.2 and 0.8 g/L of ferulic acid with a conversion rate of 80% at 0.2 g/L of ferulic acid. The conversion of ferulic acid to vanillin reduced significantly to less than 20% when the substrate concentration is higher than 0.8 g/L. To ensure high conversion rate, it is worth improving the substrate resistance of the natural host by domestication. By continuously sub-cultured in the medium with high concentrations of ferulic acid, an evolved *B. subtilis* B7 showed significantly enhanced tolerance to ferulic acid and the molar transformation rate reached 24.75% when the initial substrate concentration was 1.0 g/L.

The natural chassis may be difficult to accumulate vanillin, which is often degraded to by-products such as vanillic acid or vanilla alcohol, resulting in low conversion rates. To overcome these, pathway engineering and mutant screening techniques have been used to develop efficient production chassis. For example, vanillin dehydrogenase (VDH) was characterized as the key enzyme of vanillin degradation [24]. The function of VDH was abolished via an insertion in the corresponding gene *vdh*, achieving a conversion of 6.5 mmol/L eugenol to 2.9 mmol/L vanillin [25]. Similar work was conducted on *Pseudomonas fluoresceum* BF13, further optimizing the culture conditions and biotransformation parameters enhanced the vanillin titer to 8.41 mM (approximately 1.28 g/L), which was the highest titer in *Pseudomonas* [15]. Nevertheless, vanillin degradation was still observed due to the presence of other vanillin dehydrogenases with similar activities. To address this, vanillin biosensors have been employed to identify the mutants with accumulated vanillin [26]. Despite of these advances, it is still challenging to systematically optimize the natural hosts due to the poorly understood genetic backgrounds and limited genetic modification tools. Additionally, the biosafety is also an important issue that needs to be taken into consideration for food application.

### Developing and optimizing the recombinant chassis

To address the challenges associated with natural chassis, an alternative strategy is the development of recombinant chassis using common and well-characterized strains such as *E. coli* [27] and yeast [28]. These model organisms have well-established metabolic networks and efficient gene editing systems, making them more amenable to genetic engineering. Initial step for constructing a recombinant chassis involves introducing the desired biosynthetic pathway into the host cell. The next step is to rewire the metabolism to enhance the performance in bioproduction, including improving the strain resistance toward substrate and product as well as eliminating the byproduct formation.

Generally, the recombinant chassis is more vulnerable to substrate and vanillin due to the toxicity of these compounds. A feasible strategy is to convert vanillin into non-toxic or less toxic derivatives by introducing one or more exogenous genes into the chassis cells. In the case of yeast, 0.5 g/L vanillin significantly impeded the growth of *Schizosaccharomyces pombe* and thereby hindered the final yield [28]. By introducing a glycosylation step to convert vanillin to less toxic vanillin β-D-glucoside, the product accumulation was effectively increased. Another approach is using high-throughput screening to obtain mutants that are resistant to the product. Yoon et al. utilized NTG (N-methyl-N’-nitro-N-nitrosoguanidine) for chassis mutagenesis and obtained a vanillin-resistant mutant, NTG-VR1, successfully improved the vanillin yield by two-fold [29]. Genome sequencing along with reverse engineering could further improve the stability of the recombinant chassis in metabolic engineering. Single-gene knockout-based [30] or pooled CRISPR interference-assisted genome-scale screening [31] might also be useful for identifying mutants with superior performance.

Similar to the natural host, product instability is also a critical issue in the recombinant host. In most cases, enzymes that are responsible for vanillin degradation are not well understood, making it challenging to precisely target the genes encoding them. To this end, genome mining was conducted in *Corynebacterium glutamicum* and NCgl0324 was characterized as the enzyme with aromatic aldehyde reductase activity [32]. Knocking out the corresponding gene led to a titer of 0.31 g/L vanillin in the recombinant chassis. Similarly, a genome-wide search of all possible candidates was performed in *S. cerevisiae* and alcohol dehydrogenase gene *ADH6* was identified as the key one for vanillin degradation. Knocking out *ADH6* successfully decreased the conversion rate of vanillin to vanillyl alcohol by 50% [28]. It seems to be difficult to completely prevent the product degradation due to the presence and catalytic promiscuity of multiple endogenous alcohol dehydrogenases or aldehyde reductases in model hosts [33]. In this regard, multiple alcohol dehydrogenase genes including *dkgA, dkgB, yeae, yahk, yjgB*, and *yqhD* were simultaneously deleted in *E. coli* K-12 MG1655 strain, resulting in significantly reduced vanillyl alcohol and a 55-fold increase in vanillin yield [34]. To provide a microbial platform that accumulating the product, it may be worthy performing bioinformatics analysis to identify the enzymes with aromatic aldehyde reductase activity and systematically inactivate these enzymes.

Generally, it is challenging to predict unknown targets that indirectly affect the product biosynthesis. Genome-wide screening using biosensors that responsive to vanillin offers a solution for identifying potential useful targets for improved vanillin production [35]. As an example, the VanR-based whole-cell biosensor was developed to identify the targets related to biosynthetic efficiency and transmembrane transport of vanillin in *E. coli* [26]. By knocking out genes such as *mphT* that encoding vanillin influx protein, a production chassis with improved growth was obtained. Combined deletion of *mphT* along with *acs* and *ptsH*, two genes that are involved in cofactor and PEP consumption, respectively, enabled a production of 55.4 mmol/L vanillin at 26 h with a conversion rate of 90%. Rational engineering based on high-throughput approaches and CRISPR-assisted editing techniques will pave the way in increasing vanillin production in the microbial hosts.

## Building efficient biosynthetic pathways

**Fig. 2:**
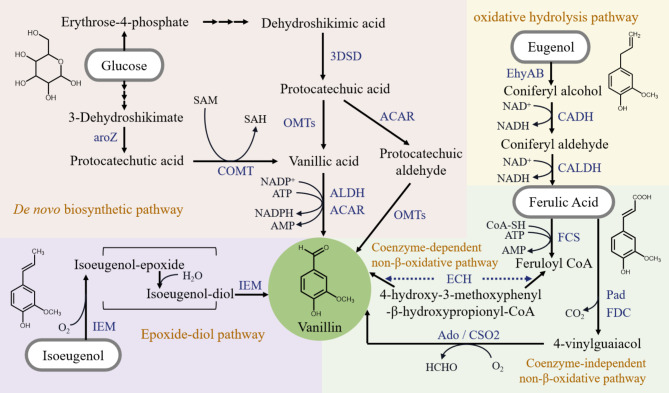
Major biosynthetic pathways of vanillin. aroZ: 3-Dehydroshikimate dehydratase(*aroZ* encoded) COMT: Catechol-O-methyltransferase (*COMT* encoded) 3DSD: 3-dehydroshikimate dehydratase(*3DSD* encoded) SAM: S-adenosylmethionine SAH: S-adenosylhomocysteine ACAR: Aromatic carboxylic acid reductase(*ACAR* encoded) OMTs: O-methyl-transferase enzymes(*OMTs* encoded) ALDH: Aryl aldehyde dehydrogenase EhyAB: Eugenol hydroxylase(*ehyA* and *ehyB* encoded) CADH: Coniferyl alcohol dehydrogenase(*calA* encoded) CALDH: Coniferyl aldehyde dehydrogenase(*calB* encoded) FCS: Feruloyl-CoA synthetase(*fcs* encoded) ECH: Enoyl-CoA hydratase/aldolase(*ech* encoded) Pad: Phenolic acid decarboxylase(*BcPad* encoded) Ado: Aromatic dioxygenase(*Ado* encoded) FDC: Ferulic acid decarboxylase(*fdc* encoded) CSO2: carotenoid cleavage oxygenase(*COS2* encoded)

Selection and engineering of the biosynthetic pathways significantly affect the final yield in biosynthesizing the target product. Several pathways have been developed for the microbial production of vanillin, and each pathway contains certain advantages and limitations (Fig. 2). To achieve industrial-scale production, it is necessary to further optimize these pathways using diverse strategies.

### ***De novo*** biosynthesis

Bioprospecting and utilizing pathways from inexpensive feedstock offer a cost-effective strategy for producing bio-based vanillin. For example, glucose is a commonly used inexpensive carbon source that could be efficiently utilized by microbial hosts. It is non-toxic to microorganisms and does not affect the flavor of the product, making it an attractive substrate for vanillin production. The *de novo* synthetic pathway in Vanilla plant consists of the phenylpropanoid pathway for synthesizing ferulic acid from glucose and a hydratase/lyase type enzyme designated vanillin synthase (VpVAN) for converting ferulic acid to vanillin glycoside [36]. When co-expressed VAN with a UDP-glucosyltransferase (UGT) from *Arabidopsis thaliana* (AtUGT72E2), vanillin glycoside formation was observed in yeast by administrating with ferulic acid. Nevertheless, the practical application of VAN-based *de novo* synthetic pathway is largely limited owing to the requirement of UGT, which is difficult to be expressed and folded in prokaryotes.

To that end, studies have focused on assembling the *de novo* synthetic pathway in microbial hosts via the integration of plant-derived and microbial-derived pathways. Ni et al. established a novel *de novo* synthetic pathway in *E. coli* by combining the plant-based phenylpropanoid pathway with the microbial-based coenzyme-dependent non-β-oxidative pathway [37]. The reconstructed pathway explored the utilization of various low-cost substrates such as tyrosine, glucose, xylose, and glycerol, producing vanillin with a titer range of 13.3 mg/L to 97.2 mg/L. This strategy provided a new insight for the construction of artificial *de novo* synthetic pathways.

By taking advantage of the native shikimate pathway of *E. coli*, Li et al. developed a novel *de novo* synthetic pathway of vanillin by driving the flux through protocatechuic acid [38]. Using this pathway, 65 mg/L and 45 mg/L of vanillin were accumulated in genetically engineered yeast strains, *S. pombe* and *S. cerevisiae*, respectively [28]. To prevent the degradation of aldehydes to their corresponding alcohols, a strain of *E. coli* K-12 MG1655 that knocking out three genes encoding aldo-keto reductases and three genes encoding alcohol dehydrogenases was obtained [34]. By transforming the *de novo* synthetic pathway into this strain, 119 mg/L vanillin was produced from glucose. Despite of these advances, the conversion rate of *de novo* synthetic pathways remains unsatisfactory.

### Biosynthesis from intermediate metabolites

One of the most commonly used pathways is the coenzyme-dependent non-β-oxidative pathway consisting of two enzymes, feruloyl-CoA synthetase (FCS) and enoyl-CoA hydratase/aldolase (ECH) (Fig. 2). In the natural hosts such as *Pseudomonas fluorescens*, *Pseudomonas putida*, *Streptomyces*, and *Aspergillus Niger*, this pathway was utilized to convert ferulic acid to vanillin, which serves as an intermediate metabolite [39]. The advantage of this pathway lies in the simplicity of construction and particularly the efficiency of catalysis due to the participation of cofactors. However, it has several limitations such as dependence on expensive cofactors, tendency to produce by-products, and the relatively high cost of ferulic acid (US $ 180/kg).

While cofactor dependence of FCS-ECH pathway could be partially addressed by increasing the supply of cofactors, the continued demand for expensive coenzymes may result in a substantial loss of value of this process. The utilization of novel enzymes that do not require coenzymes could overcome this problem while ensuring economic viability. To this end, genome mining was performed to identify enzymes that catalyzing the second step, leading to the characterization of a new coenzyme-independent oxygenase, CSO2 [40]. This enzyme cleaves the carbon-carbon double bond of 4-vinylguaiacol to form vanillin. By combining this enzyme with the existing decarboxylase, FDC, a coenzyme-independent two-step pathway was constructed in *E. coli* [41]. By designing a two-stage production process with the whole-cell catalyst, 7.8 g/L vanillin was produced from ferulic acid through 4-vinylguaiacol with a conversion rate of 69.3%.

Other vanillin biosynthetic pathways included the oxidative catabolic pathway and the epoxide-diol pathway that start from eugenol and isoeugenol, respectively. The oxidative catabolic pathway has been characterized in bacteria such as *Pseudomonas sp*. HR199 [25]. It utilizes eugenol as the substrate, which is catalyzed by eugenol hydroxylase (encoded by *ehyA* and *ehyB*), coniferyl alcohol dehydrogenase (encoded by *calA*), and coniferyl aldehyde dehydrogenase (encoded by *calB*) to form ferulic acid and then converted to vanillin [42]. Although eugenol (US $ 20/kg) is much cheaper than ferulic acid, the yield using this pathway is less than 1 g/L probably due to the requirement of multiples enzymes [43]. An epoxide-diol pathway that transforming the eugenol isomer, isoeugenol, to vanillin via oxidation of the side chains of isoeugenol has been revealed in bacteria such as *Bacillus* [44]. The key enzyme in this pathway is isoeugenol monooxygenase (IEM), which is encoded by the gene *IEM* for the conversion of isoeugenol to vanillin [45]. Microbial hosts that naturally harboring this pathway have been widely explored in vanillin production due to the high yield and the cheap cost of isoeugenol (US $ 23–26/kg). The practical application of recombinant *E. coli* overexpressing IEM has also been investigated by systemically optimizing the conditions of enzyme production and whole-cell catalysis process [46].

## Host engineering

### Enzyme bioprospecting and engineering

**Fig. 3:**
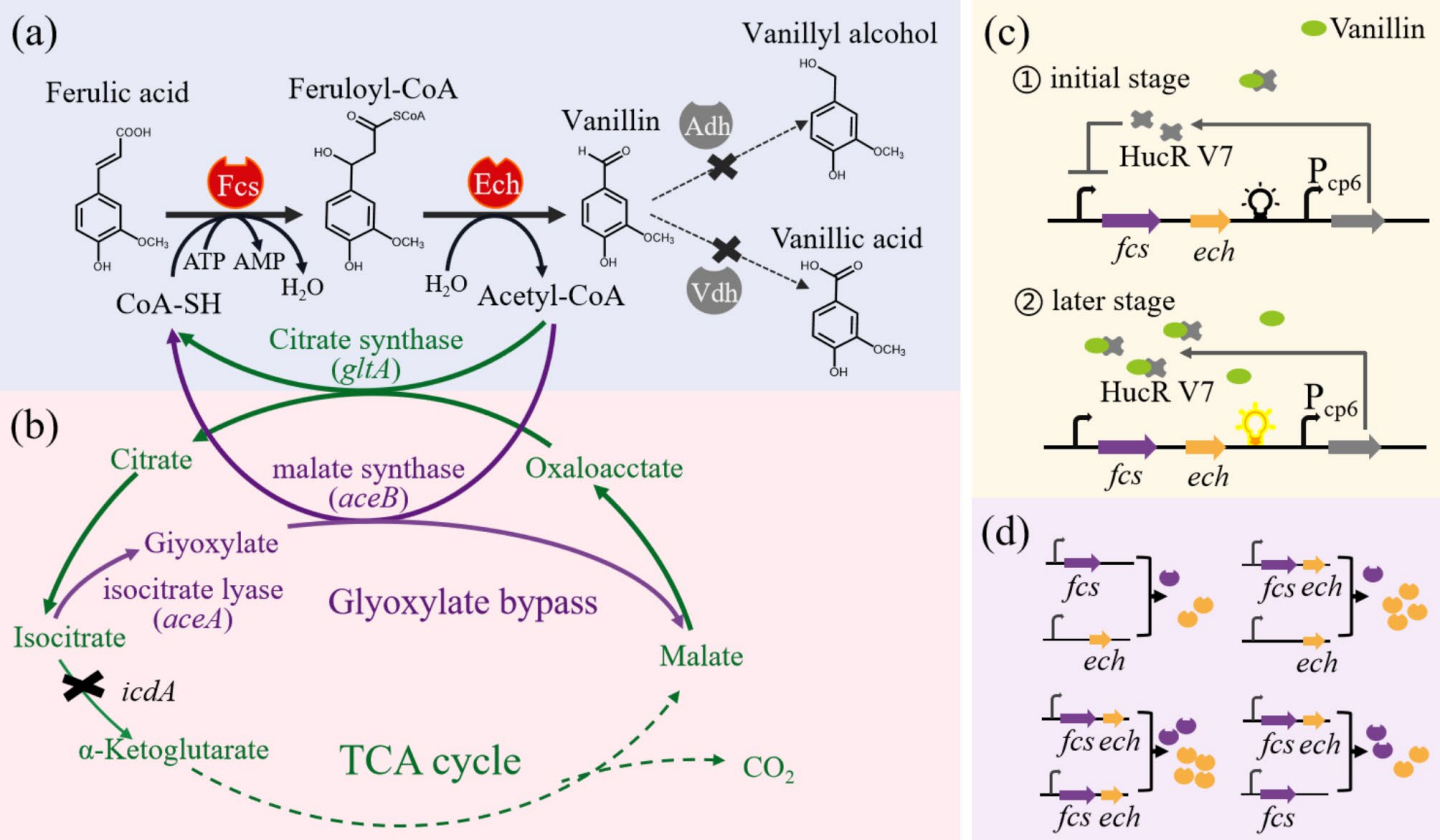
Strategies to optimize FCS-ECH pathway for vanillin production. (**a**) Mining of robust enzymes. (**b**) Cofactor engineering. (**c**) Dynamic control of vanillin biosynthesis by an artificial regulatory circuit. (**d**) The expression level of key enzyme genes in the pathway is fine-tuned

Enzymes play crucial roles in determining the efficiency of metabolic pathways. By exploring biosynthetic enzymes with unique properties such as thermostability and pH stability, it is possible to improve the conversion efficiency and reduce vanillin degradation under certain defined conditions (Fig. 3a). To this end, two thermostable enzymes that screened out from a thermophilic actinomycete *Amycolatopsis thermoflava* N1165, including AtFCS and AtECH, were introduced to establish the coenzyme-dependent non-β-oxidative pathway in *E. coli*, generating a temperature-directed whole-cell catalyst for vanillin biosynthesis [47]. The catalysts produce 1.1 g/L of vanillin within 30 min and significantly lower the level of by-products at a reaction temperature of 50 ℃, under which the activities of endogenous alcohol dehydrogenases (ADHs) were reduced. It should be noted that vanillyl alcohol still occupied 23.5% in the final products, this could be attributed to the remaining activities of ADHs in the whole-cell catalysts. Searching for highly thermostable enzymes may further enhance the practicality of this strategy.

The low catalytic activity and stability of the rate-limiting enzyme CSO2 greatly hindered the utilization of coenzyme-independent pathway. To address this, Yao et al. designed site-directed mutagenesis strategies to improve the activity and thermal stability of CSO2, yielding mutants that facilitating up to 62% increase of vanillin production [48]. To break the catalytic limit of CSO2, Ni et al. performed bioprospecting to identify efficient enzymes for aromatic olefins oxidation. An aromatic dioxygenase (Ado) with catalytic efficiency 78,500-fold higher than COS2 was identified from thermophilic fungus *Thermothelomyces thermophila*, efficiently converting 4-vinylguaiacol to form vanillin. Moreover, the phenolic acid decarboxylase (Pad) that catalyzing nonoxidative decarboxylation of ferulic acid to 4-vinylguaiacol was also characterized from thermophilic bacterium, *Bacillus coagulans* DSM1 [49]. Then the two enzymes were employed to establish a temperature and pH-directed whole-cell catalyst, which serves as a versatile platform for the production of the lignin-derived aromatic aldehydes in a coenzyme-independent manner.

### Cofactor engineering

Biosynthetic pathways such as the coenzyme-dependent non-β-oxidative pathway utilize CoA and ATP as the cofactors and released acetyl-CoA as the byproduct, thereby recycling the cofactors can be beneficial for the continuous production (Fig. 3b). To improve the supply of CoA, Lee et al. enhanced the expression levels of citrate synthase and malate synthase, both enzymes catalyze the reactions that consuming acetyl-CoA to CoA [38]. The resulting strain accumulated as high as 5.14 g/L of vanillin by using a resin that adsorbing vanillin to reduce the toxicity. Another strategy is to block the reaction that consumes CoA. It has been documented that deleting the *acs* gene of acetyl-CoA synthetase, which catalyzes the irreversible conversion of acetate to acetyl-CoA by consuming ATP, was effective in supplying CoA and ATP [26]. It is worth testing the synergistic effect of these cofactor engineering strategies for improved vanillin production.

### Establishing biosensors for high-throughput screening

Compared with GC/MS, LC/MS and other conventional methods that are time-consuming, biosensor-assisted detection method enables efficient and rapid in situ screening for strain mutants with increased level of target products. To this end, developing the vanillin-responsive biosensor is of highly interest. By applying computational design and in vitro prototyping, several vanillin-responsive effectors were obtained from the variants of a tetR family repressor, and one of the effectors showed nearly three-fold change of fluorescence at a concentration range from 0 to 1 mmol/L [50]. This effector could be employed to design a dynamic feedback circuit by regulating the expression of vanillin efflux pump or enzymes that converting vanillin to fewer toxic compounds. Sana et al. developed a modified *E. coli* biosensor using a vanillin-inducible promoter and optimized the detection limit of vanillin to as low as 200 µmol/L [50]. This biosensor has been used to screen new lignin-degrading enzymes capable of converting lignin to vanillin. Similarly, a whole-cell biosensor that selectively detecting vanillin in a linear and wide range was established by using transcription regulators and promoters of the *E. coli* emrRAB operon, enabling the rapid and functional screening of lignin-converting enzymes from the metagenomes of uncultivable archaea and bacteria [51].

### Fine-tuning the expression level

Optimizing metabolic network is important for the development of a stable and efficient bioproduction system. Dynamic regulation has emerged as an effective tool in managing the trade-off between cell growth and production in metabolic engineering [52]. To realize the regulation of metabolic flux in vanillin biosynthesis, Liang et al. performed directed evolution to obtain a regulatory component that is responsive to ferulic acid and vanillin with distinct capacities [53]. This component facilitated the temporal control of enzymes expression, allowing low levels of pathway expression for better growth at the early stage and activated pathway expression for high production in late stage (Fig. 3c). By eliminating product toxicity and cell burden, the multi-layer dynamic control strategy effectively balanced cell growth and product formation. Another strategy is regulating metabolic flux via fine-tuning the ratio of biosynthetic enzymes (Fig. 3d). By investigating the optimal amount of vanillin biosynthetic enzymes in a cell-free system and adjusting the ratio of FCS and ECH with regulated copy number, a recombinant *E. coli* with improved synthetic rate was obtained [43]. The resulting whole-cell catalyst was capable of converting 20 mM ferulic acid to 15 mM vanillin within 6 h.

## Optimizing the bioconversion process

### Whole-cell catalysis

Whole-cell catalysis using resting cells possesses the advantages of shorter time-consuming, easier separation of products, and higher substrate conversion rate compared with the conventional fermentation. In contrast to the cell-free system, whole-cell catalysis eliminates the complicated purification process, thereby the enzymes are still in the original state of living cells and the intracellular multi-enzyme system can easily realize the enzyme cascade reaction. For vanillin production, whole-cell catalysis exhibits strong reaction specificity with improved conversion rate by avoiding the detrimental effect of substrates and vanillin to cell growth [21]. After optimizing the enzyme production and whole-cell catalysis process, the recombinant *E. coli* expressing IEM from *Pseudomonas nitroreducens* Jin1 achieved a vanillin productivity of 115 g/L/d in 82.3% yield [46]. To highlight the potential, the whole-cell catalysis technology could be extensively applied along with other condition optimization strategies [43].

### Enzyme immobilization

Enzyme immobilization is a vital engineering technique that bind free enzymes to a specific space or insoluble carrier to restrict their mobility. The interaction with the immobilized substrate may alter the spatial structure of the enzyme outside the catalytic active center, thereby improving the enzyme stability in harsh environments, such as strong pH, high temperature, or organic solvents. Target product could be obtained simply by mixing the substrates with the immobilized enzymes, which could be reused in successive catalytic reaction cycles. The production of vanillin using immobilized enzymes is worthy of exploring especially for the simple synthetic pathways. To solve the instability problem of CSO2 and to simplify the process, COS2 was immobilized on Sepabeads EC-EA anion exchange carriers, enabling the production of 6.8 mg vanillin from isoeugenol at a 1 mL scale over 10 cycles of reaction [54]. When FDC and CSO2 were immobilized simultaneously, the system was able to synthesize 2.5 mg vanillin from ferulic acid after 10 reaction cycles. This is the first case of vanillin production using immobilized enzyme biotechnology. However, the low catalytic activity of the CSO2 and the accumulation of 4-vinylguaiacol necessitates the addition of a large amount of enzyme to the reaction system, hampering the system efficiency and limiting the final yield. It is worth testing the immobilized effect of efficient alternatives of CSO2 in the production of vanillin.

### Microbial fermentation process

For conventional fermentation process, it is necessary to systematically explore and optimize the microbial fermentation parameters. To enhance the commercial viability of vanillin production strains, various approaches have been employed, such as optimizing cultivation conditions, adjusting feeding strategies, and implementing a phased recovery technique.

#### Optimizing fermentation conditions

Numerous factors such as medium composition, pH, temperature, and dissolved oxygen could significantly influence the cell growth and the bioconversion process. When aiming to improve vanillin production, it is recommended to evaluate the effect of each fermentation parameter individually and then calculate the influence of multiple factors using the orthogonal matrix method. Using this strategy, the fermentation conditions of the wild fungus *Pycnoporous cinnabarinus* were optimized and the molar conversion rate of ferulic acid to vanillin was successfully increased by four-fold [17]. Similarly, the factors influencing the conversion of ferulic acid to vanillin was investigated for an isolated *Bacillus subtilis* strain, including temperature, pH, initial substrate concentration, and inoculum size [55]. By conducting repeated small-batch experiments in optimized conditions, a conversion efficiency of 63.30% was achieved after 3-h transformation.

#### Feeding precursors

Ferulic acid, commonly used substrate in vanillin production, exhibits obvious toxicity to bacteria due to the antibacterial properties. Since the concentration of ferulic acid in the medium directly affects the yield of vanillin, controlling the concentration in the fermentation broth is essential to achieve a trade-off between the yield and toxicity. To this end, the fed-batch production technique by implementing a multi-pulse feeding strategy is recommended to enhance the overall yield of vanillin. When ferulic acid was supplemented in batches through three rounds of pulses, the native-producer *Amycolatopsis sp.* ATCC 39,116 reached 0.46 g/L/h vanillin with a maximum titer of 7.76 g/L [56]. It should be noted that the precursor feeding strategy needs to be combined with strategies that eliminate product toxicity, which is easily caused by vanillin accumulation in the fermentation broth.

#### Product recovery

Recovery of vanillin by in situ adsorption using macroporous resins [56] or magnetic chitosan membrane [46] could alleviate the threat of vanillin accumulation to cell. In situ adsorption using resins has been widely used and the choice of resin and its dosage directly affect the overall adsorption effect. In this regard, the resin effect to each fermentation system should be individually explored for achieving the highest vanillin yield. For example, 50% (w/v) of XAD-2 resin has been proved to be effective in removing vanillin from the culture medium, obtaining 2.9 g/L vanillin from 10 g/L ferulic acid and twice the yield in the absence of resin [29]. Among several macroporous adsorption resins that were selected for vanillin adsorption during the bioconversion of *Streptomyces sp*. strain V-1, 8% DM11 resin (w/v) exhibited the best performance by enabling 19.2 g/L vanillin production from 45 g/L ferulic acid within 55 h [16]. Because the adsorbed vanillin needs to be purified with several steps, the recovery process will bring certain loss in the total vanillin.

## Perspectives

With the expansion of growing demand, the global vanillin market is experiencing a substantial increase in recent years, reaching as high as USD 886.5 million [57]. Currently, most vanillin in the market is produced by chemical synthesis, including petroleum-based (85%) and lignin-based (15%) ones. However, the aromatic intensity especially that of petroleum-based vanillin is unsatisfactory and the chemical production process is not preferred due to its unsustainability. Vanillin from vanilla extract satisfies the consumer demand for natural products but is constrained in applications due to the limited supply and the relatively high prices. Biomanufacturing using genetic engineering, cell engineering, enzyme engineering, fermentation and whole-cell catalysis offers renewable solutions by alleviating the oil resources crisis, thus is preferred in establishing a carbon-neutral society. Major flavor enterprises have moved forward to use advanced biotechnology to produce bio-based flavors, which harbor the same aroma and taste as the natural ones without altering the nutritional value. The rising perception of bio-based flavors drives the consumer demand toward bio-based vanillin, which exhibits diversified functionality in end-use industries, such as food and beverage, pharmaceutical, personal care, animal and poultry feed, and packaging industries.

**Table 1:** Summary of vanillin production from different feedstocks

Substrate	Microorganisms	Scale	Titer	Yield	Productivity	References
Ferulic acid	*B. subtilis* B7-S	Bioreactor	379 mg/L	0.63 g/g ferulic acid	/	[55]
	*P. fluorescens* BF13-1p4 with pBB1	Bioreactor	1.28 g/L	84.1% (molar yield)	/	[15]
	*Streptomyces sp.* V-1	Bioreactor	19.20 g/L	0.43 g/g ferulic acid	/	[16]
	*E. coli* DH5α with pTAHEF	Bioreactor	1.12 g/L	0.78 g/g ferulic acid	/	[58]
	*E. coli* NTG-VR1	Flask	2.90 g/L	0.29 g/g ferulic acid	/	[29]
	*E. coli* JM109-FE-F	Bioreactor	2.28 g/L	75.0% (molar yield)	0.126 g/L/h	[43]
	*E. coli* DH5α (Δ*icdA*) with pTAHEF-*gltA*	Bioreactor	5.14 g/L	86.6% (molar yield)	/	[38]
	*E. coli* VA1 with pETDuet-*fcs*-*ech*	Bioreactor	/	86.7% (molar yield)	1.10 g/L/h	[49]
	*Amycolatopsis sp.* ATCC 39,116	Bioreactor	7.76 g/L	0.69 g/g ferulic acid	0.46 g/L/h	[56]
	*Amycolatopsis sp.* ATCC 39,116	Flask	9.18 g/L	96.1% (molar yield)	/	[59]
	*Amycolatopsis sp.* ATCC 39,116 Δvdh::permE*::echfcs with utilization of resin	Bioreactor	22.30 g/L	/	/	[60]
	*Streptomyces* V1	Flask	9.09 g/L	95.2% (molar yield)	/	[59]
Eugenol	*Bacillus safensis* SMS 1003	Bioreactor	120 mg/L	26.0% (molar yield)	/	[14]
	*P. resinovorans* SPR1	Bioreactor	240 mg/L	10.0% (molar yield)	/	[22]
	*Pseudomonas sp.* HRvdhΩKm	Bioreactor	441 mg/L	44.6% (molar yield)	/	[25]
Isoeugenol	*B. pumilus* S-1	Bioreactor	3.75 g/L	40.5% (molar yield)	/	[18]
	*Pseudomonas sp.* ISPC2	Bioreactor	1.15 g/L	12.4% (molar yield)	/	[19]
	*Psychrobacter sp.* CSW4	Bioreactor	1.28 g/L	/	/	[61]
	*Candida galli* PG06	Bioreactor	1.12 g/L	25.7% (molar yield)	/	[20]
	*Trichosporon asahii* MP24	Bioreactor	2.40 g/L	52.5% (molar yield)	/	[21]
	*Bacillus fusiformis* CGMCCl347	Bioreactor	3.25 g/L	/	/	[44]
	*Bacillus fusiformis* CGMCC1347 with utilization of HD-8 resin	Bioreactor	8.10 g/L	0.29 g/g Isoeugenol	/	[62]
	*Bacillus fusiformis* CGMCC1347 with utilization of HD-8resin after 6 times reuse of immobilized cells	Bioreactor	39.26 g/L	/	/	[63]
	*E. coli* BL21(DE3) with pET-21a-*iem*	Bioreactor	/	0.76 g/g Isoeugenol	115 g/L/d	[46]
Glucose	*S. cerevisiae* VAN286	Bioreactor	65 mg/L	/	/	[28]
	*S. pombe* VAN294	Bioreactor	45 mg/L	/	/	[28]
	*E. coli* K-12 MG1655	Bioreactor	119 mg/L	/	/	[34]
	*C. glutamicum* PV-IYΔ0324	Flask	0.31 g/L	/	/	[32]
	*E. coli* BL21(DE3) VT-4	Bioreactor	19.3 mg/L	1.93 mg/g Glucose	/	[37]

Currently, bio-based vanillin could be obtained from various feedstocks, including eugenol, isoeugenol, ferulic acid and glucose (Table 1). Eugenol is present in many natural essential oils, among which clove oil, bay leaf oil and clove basil oil contain high levels of eugenol. Although bio-based vanillin that producing from eugenol is less than 1 g/L scale, it is favored by the high-end food and beverage market with a strong aroma and high safety. Indesso Aroma company in Indonesia produces vanillin at a large scale through this method probably because Indonesia is the world’s leading producer of clove oil. Ferulic acid is one of the most abundant phenolic compounds, which is widely found in wood fibers and plant cell walls. The process of converting ferulic acid to vanillin is simple with advantages of high efficiency, low pollution and high food safety. The highest titer has reached as high as 22.30 g/L, which is close to the industrial scale. Glucose is the most commonly used substrate in metabolic engineering due to its cheap price, non-cytotoxicity in large-scale fermentation. Because natural microorganisms are unable to metabolize glucose to vanillin, studies have mainly focused on the engineering of recombinant hosts. Despite of the advances, the maximum yield only reached as low as 310 mg/L, which was probably caused by the long steps and low performance of enzymes. The emergence of data-driven approaches such as artificial intelligence (AI)-assisted pathway design strategies provides opportunities for advanced pathway and enzyme design [64]. It is worth exploring the biosynthetic pathways that starting from cheap glucose-yielded substrates via artificial design.

The commercial feasibility and technical economy are critical issues in the bioproduction of vanillin because companies with high production capacities and low costs could gain a competitive advantage. These could be realized by utilizing cheaper substrates and increasing the bioconversion rate. As a representative vanillin market leader, Solvay established a biotechnology platform in May 2022 to provide sustainable solutions for vanillin markets. By valorizing a byproduct of rice bran, Solvay successfully launched new natural vanillin flavors using a cheap substrate and a fermentation process in September 2022, demonstrating the commercial feasibility of microbial production of vanillin. By further increasing the bioconversion rates and decreasing the feedstock costs, the bio-based vanillin produced from generally regarded as safe (GRAS) hosts will become worldwide commercially available.

In this review, we explored and highlighted the potential of biotechnology for vanillin production. By summarizing the current progress of vanillin bioproduction technologies, we addressed the main bottlenecks in this area and provided potential approaches for improved production from the perspectives of chassis, pathway and fermentation. It is highly expected that the maturation of bioproduction technology using inexpensive raw materials and GRAS hosts will be of great significance to the global flavor and fragrance market.

## Data Availability

All data and materials in this study are included in this published article.
